# Premature Ventricular Contraction-Induced Cardiomyopathy: Contemporary Evidence from Risk Stratification, Pathophysiology, and Management

**DOI:** 10.3390/jcm13092635

**Published:** 2024-04-30

**Authors:** Tanawat Attachaipanich, Ben Thiravetyan, Narisara Tribuddharat, Surachat Jaroonpipatkul, Leenhapong Navaravong

**Affiliations:** 1 Cardiac Electrophysiology Research and Training Center, Chiang Mai University, Chiang Mai 50200, Thailand; tanawat.att@cmu.ac.th; 2 Department of Immunology, Faculty of Medicine Siriraj Hospital, Mahidol University, Bangkok 10700, Thailand; ben.thi@mahidol.edu; 3 Vichaiyut Hospital, Bangkok 10400, Thailand; n.tribuddharat@gmail.com; 4 Division of Cardiology, Rajavithi Hospital, College of Medicine, Rangsit University, Bangkok 10400, Thailand; surachat.ja@rsu.ac.th; 5 Intermountain Heart Institute, Utah Valley Hospital, Provo, UT 84604, USA

**Keywords:** premature ventricular complex, cardiomyopathy, catheter ablation, antiarrhythmic drug

## Abstract

Premature ventricular complexes (PVCs) are commonly encountered problems in clinical settings. The range of symptoms can be from asymptomatic to palpitations, fatigue, or heart failure symptoms. A higher burden of PVCs is a risk factor for development of PVC-induced cardiomyopathy (PIC). Rhythm evaluation by 12-lead ECG and an ambulatory monitoring device are essential. Currently, several imaging modalities, such as echocardiography and cardiac magnetic resonance imaging, are utilized to evaluate the underlying structure that may be related to PIC. Beta blockers and antiarrhythmic drugs are typically part of the initial management strategy. If these fail, catheter ablation of PVCs is typically the next step. The purpose of this article is to summarize the current evidence/knowledge about PIC.

## 1. Introduction

Premature ventricular complexes (PVCs) were traditionally deemed benign in the absence of underlying structural heart disease with therapeutic interventions reserved for alleviating symptoms [1]. However, recent research has illuminated the enduring effects of frequent PVCs, underscoring their involvement in contributing to cardiomyopathy and symptoms of heart failure [2,3].

The diagnosis of PVC-induced cardiomyopathy (PIC) relies on identifying frequent PVCs, an existing cardiomyopathy, and excluding alternative causes. Idiopathic PIC is diagnosed when no other etiology for frequent PVCs is identified. Notably, in patients with established cardiomyopathy from other heart conditions, such as coronary artery disease, frequent PVCs can worsen the existing condition. Advanced imaging techniques, such as cardiac magnetic resonance imaging (CMR), can unveil the extent and etiology of cardiomyopathy [4].

Over the past two decades, catheter ablation of PVCs has evolved. Technological advancements, including 3D mapping systems, have revolutionized the precision of electroanatomic mapping during ventricular tachycardia (VT) ablation, marking significant progress in the field [5,6]. This article explores the risk factors associated with PIC and delves into available treatment options.

## 2. Risk Factors

One of the most significant risk factors of PIC is the PVC burden. Numerous studies have demonstrated an association between PVC burden and PIC [7,8,9,10,11,12,13,14,15,16]. However, the specific cutoff point of a PVC burden capable of inducing cardiomyopathy remains unknown. Moreover, details of PVC characteristics, including PVC origin site and PVC QRS duration, could also influence the threshold of PVC burden to develop PIC [9,16]. Various studies focusing on the absolute number of PVCs have reported that a burden exceeding 10,000 to 20,000 per day is associated with PIC [7,9,12]. It is important to note that most of these studies are cross-sectional, limiting their implications for establishing causal relationships due to the nature of their study design [9,12].

A prospective study aiming to determine the impact of PVC burden, which included patients with frequent PVC, defined as more than 1000 per day, reported that a PVC burden exceeding 20,000 per day was associated with both a decline in left ventricular ejection fraction (LVEF) and an increase left ventricular end-diastolic diameter (LVEDd) over a mean follow-up period of 5.6 years [7]. Instead of considering the absolute number of PVCs, some studies assess PVC burden as a percentage. A PVC burden of 16–24% was reported as a predictor for cardiomyopathy in several cross-sectional studies [8,9,11,14]. In a study involving patients undergoing radiofrequency ablation (RFA), a PVC burden exceeding 24% was identified as a predictor of impaired LVEF, with a sensitivity of 79% and specificity of 78% [11].

While many studies have demonstrated a cross-sectional relationship between PVC burden and a decline in LVEF, several reports indicate a reduction in PVC burden after RFA, leading to an improvement in LVEF and LVEDd during follow-up [8,10,13,17]. Furthermore, in patients with a normal LVEF assessed using conventional echocardiography, speckle tracking imaging has reported reduced left ventricular (LV) and right ventricular (RV) strain [18]. Post-ablation of PIC was not only associated with an improvement in systolic dysfunction but also demonstrated improved LV diastolic dysfunction and left atrial (LA) remodeling, as evidenced by improvements in the E/A ratio and LA volume index [19]. These results suggest a potential causal relationship between PVC burden and cardiomyopathy. Although PVC burden is a crucial contributing factor to cardiomyopathy, some patients with a high burden do not develop cardiomyopathy, while others with a low PVC burden do develop cardiomyopathy [20,21]. This indicates the presence of other factors contributing to cardiomyopathy.

Regarding the site of PVC origin, most studies have reported PIC by including patients with PVC originating from the right ventricular outflow tract (RVOT) [8,12,17]. However, the site of PVC origin may have a varying impact on PIC. A cross-sectional study involving symptomatic frequent PVC patients who underwent RFA demonstrated that the threshold for PVC burden leading to LVEF decline differed between right and left ventricular PVC, being 10% and 20%, respectively [9]. Furthermore, other studies have reported that PVC with epicardial origin and non-outflow tract were independent predictors of cardiomyopathy [16,22].

Certain characteristics of PVC may be stronger predictors of PIC than others. PVC with a QRS duration longer than 150 milliseconds (ms) has been linked to cardiomyopathy [16,22]. Broad PVC QRS was associated with a lower PVC burden threshold for cardiomyopathy [16]. In a retrospective study involving patients with frequent PVC defined as a PVC burden of more than 10% per day, who underwent RFA with normal baseline LVEF, a PVC QRS duration ≥ 153 ms was identified as an independent predictor of LV dysfunction, regardless of PVC burden, with a median follow-up time of 14 months [22]. These results could be explained by the impact of dyssynchronous LV contraction on cardiomyopathy. Another parameter, including PVC interpolation, has also shown an association with LV dysfunction [13]. The evidence regarding the effect of the coupling interval on PIC is still conflicting. However, a study in a canine model indicated that a longer coupling interval of PVC was associated with greater LV dyssynchrony [23]. In a clinical study, one cross-sectional study in patients referred for ablation due to PVC-associated symptoms demonstrated that there was no association between the coupling interval and lower LVEF [9]. Another study in asymptomatic children with PVC reported an association between a shorter coupling interval and lower LVEF [24]. However, these studies included only a limited number of participants.

Not only do PVC characteristics affect the development of PIC, but patient characteristics themselves also play a role. Sex impacts both the prevalence and prognosis of certain cardiac arrhythmias [25,26]. Some types of arrhythmia are more common in males, such as Brugada syndrome, while others are more common in females, such as long QT syndrome [25]. Male sex has been reported to be associated with a higher prevalence of PVC [27,28]. A report from a large cohort study, the Atherosclerosis Risk in Communities (ARIC) study, which had 15,792 participants, found that male sex was associated with an increased risk of PVC [28]. Several retrospective and prospective studies have demonstrated that being male is a predictor of PIC [29,30,31]. A retrospective study involving 1185 patients who underwent PVC ablation reported that male sex was an independent predictor for the development of PIC [30]. While the impact of sex hormones on ion channels has been studied, the exact mechanism of sex on PIC development remains unclear, and further studies are needed [25].

Further longitudinal studies for risk stratification in PVC patients are required to provide optimal treatment strategies for preventing LV dysfunction in the appropriate population. The clinical studies on PVC burden, characteristics, and PIC are comprehensively summarized in Table 1.

## 3. Pathophysiology

PIC was initially considered a manifestation of tachycardia-induced cardiomyopathy. However, evidence from several clinical studies indicates that PIC can occur without a significant difference in mean heart rate, as evaluated by 24 h Holter monitoring [7,8,12]. In an in vivo study using a swine model with bigeminy pacing compared to regular RV apex pacing at 140 bpm for 14 weeks, there was a worsened decline in LVEF along with more disturbance in myocardial calcium-handling proteins [32]. Another study utilizing a canine pacing model reported eccentric cardiac hypertrophy remodeling in the pacing ventricular bigeminy group for 12 weeks, while there was no significant change in the canine pacing atrial bigeminy group [33]. These results suggest that tachycardia and heart rate irregularity may not be major contributors to PIC.

The process of PIC is proposed to involve functional changes and is considered a reversible process. Evidence from several clinical studies has reported reversible impairment of LVEF along with LVEDd dilation after successful RFA [8,10,13,17]. A study in a canine model, pacing ventricular bigeminy at the RV apex for 12 weeks, resulted in cardiomyopathy that resolved after 4 weeks of pacing discontinuation, and pathological examination revealed no inflammation or fibrosis [34]. Another study using the same model reported an increase in the ERK1/2 and AKT/mTOR pathways, which could contribute to cardiac remodeling [35]. However, other studies using a different model in swine, pacing ventricular bigeminy at the RV apex for 12–14 weeks, reported biventricular cardiac fibrosis along with an increase in collagen type I expression [15,36]. The discrepancies in these results could be explained by differences in animal species, as well as the threshold of PVC burden and duration needed to develop cardiac fibrosis. In humans, cardiac MRI studies in PIC patients revealed that a small number of patients had late gadolinium enhancement, which could explain why some patients do not experience improvement in LV function after ablation [37].

The impaired LVEF in PIC could be a result from functional impairment involving calcium handling. Previous in vivo studies in a canine model with pacing ventricular bigeminy reported a decreased L-type calcium current (I_CaL_) based on a patch-clamp study and a decrease in junctophilin 2 expression, a dyad protein that interacts with calcium-handing proteins and ion channels, at 12 weeks [38]. Furthermore, there was an upregulation of phospholamban (PLN), downregulation of sarcoplasmic/endoplasmic reticulum Ca^2+^ ATPase-2a (SERCA2a) expression, and PLN phosphorylation [39]. Another study in a swine model with pacing ventricular bigeminy reported upregulated Ca^2+^/calmodulin-dependent protein kinase II (CaMKII-α), phosphorylated ryanodine receptor 2 (pRyR2) at the Ser 2814 CaMKII-α binding site, and downregulated SERCA2a [32]. This information indicates that LV impairment could be attributed to calcium handling impairment.

LV dyssynchrony is an acute effect of PVC that could contribute to the development of cardiomyopathy. LV dyssynchrony occurs due to abnormal myocardial activation, leading to non-simultaneous LV contraction, which could be influenced by the PVC origin. Epicardial origin of PVC causes greater LV dyssynchrony than endocardial PVC [32]. A study in a swine model reported that pacing at the LV epicardium resulted in lower LVEF and more extensive cardiac fibrosis compared to RV free wall pacing [32]. A cross-sectional study including patients referred for PVC ablation reported an association between PVC epicardial origin and LVEF decline [16]. A longer QRS duration, which impacts LV dyssynchrony, was also associated with greater LVEF impairment in both animal and clinical studies [16,32]. Moreover, a longer QRS duration was a predictor of LV function recovery post-ablation [40]. These results indicated the potential role of LV dyssynchrony in contributing to the development of PVC-induced cardiomyopathy. Further studies are needed to demonstrate the causal relationship between the acute effect of PVC, including LV dyssynchrony, and the development of PVC-induced cardiomyopathy. 

## 4. Diagnosis

Most PVC patients have a benign course with no requirement for treatment [41]. However, up to one-third of cases might develop significant symptoms and function deterioration [42]. Thus, the detection of PVCs is crucial to prevent progression into cardiomyopathy or reverse any deterioration that has already occurred.

The diagnosis of PIC is based on the presence of frequent PVCs and existing cardiomyopathy without other alternative causes. A lower PVC burden can also cause cardiomyopathy, but it is harder to confirm as the etiology and usually the resolution of cardiomyopathy after the termination of PVC is required to confirm the diagnosis [4]. Because there are many etiologies of PVCs including cardiomyopathy itself, a detailed history and adequate investigations are needed to confirm whether there are other potential causes of the PVC and cardiomyopathy [43], making PIC a diagnosis of exclusion most of the time.

PIC patients usually come to the hospital with palpitations, syncope, shortness of breath, or chest discomfort [42,43]. However, many can be asymptomatic, and the only abnormal physical examination finding may be irregular pulses from ectopic beats if they happen frequently enough. An ECG can diagnose the existence of PVCs and determine the location of ectopic foci. However, Holter monitoring, ideally for 48–72 h or even longer, is needed to estimate the PVC burden to ensure the cardiomyopathy could result from the said arrhythmia [42,44]. Studies have found that a higher frequency of PVCs correlates to the severity of LV dysfunction [11,12,45]. However, it is important to note that cases with frequent PVCs do not necessarily have cardiomyopathy and it is thought that the duration of having PVC might also correlate to the development of PIC [11,44,46,47], making a clear cutpoint that assures the development of cardiomyopathy hard to estimate. Some authors have suggested 10,000–20,000 PVCs/day or PVCs > 10% of total beats as a high PVC burden [42]. Baman et al. introduced a cutpoint of PVC burden > 24% of total beats that showed sensitivity and specificity at 79% and 78%, respectively, and Hasdemir et al. nominated a cutpoint of PVC burden > 16% with sensitivity and specificity at 100% and 87%, respectively [10,14]. One study raised the use of PVC index using PVC burden, PVC-QRS width, and epicardial origin to help identify patients with a high probability of PIC [46].

A 12-lead ECG serves as a crucial tool in localizing the likely site of origin of PVCs. This information is essential for patient counseling and treatment planning, as different PVC locations are associated with varying success rates and risks of complications from catheter ablation [4]. Predominantly, PVCs arise from the RVOT or left ventricular outflow tract (LVOT), constituting approximately two-thirds of all idiopathic cases [42]. PVCs originating from ventricular outflow tracts typically display an inferior axis, characterized by positive QRS complexes in the inferior leads and negative QRS complexes in aVR and aVL [27]. The precordial transition, the first precordial lead to display a net positive QRS complex, can be used to differentiate between RVOT and LVOT origins. A precordial transition occurring at V4 or later is suggestive of RVOT origin, while those occurring at V2 or earlier are suggestive of LVOT origin [27]. As for epicardial origin, the intermyocardial conduction delay results in a slurred initial portion of QRS complexes, which can be seen in several parameters such as prolonged pseudodelta wave, intrinsicoid deflection time, RS complex duration, or higher maximum deflection index [48,49].

A study found that PVCs with RV origins are statistically correlated to persistently reduced EF even after ablation [47]. However, other studies showed only epicardial origins are correlated with the development of PIC and are associated with the highest risk of cardiomyopathy [30]. 

A transthoracic echocardiogram should be done to estimate the degree of cardiac dysfunction and detect other structural abnormalities to ensure no other etiology for frequent PVCs. A LVEF of <50% has been considered as a cutoff to distinguish PIC from PVC patients without cardiomyopathy [50]. Echocardiogram findings that have been seen in PIC are mild to moderate decreased LVEF, increased LV systolic and diastolic dimensions, global wall motion abnormalities, and mitral regurgitation [42,44]. A global longitudinal strain is shown to be a significant mortality predictor [47].

CMR and coronary angiogram are also useful for evaluating existing fibrosis and cardiovascular diseases [43]. CMR is recognized as the gold standard for tissue characteristics’ evaluation and when used in conjunction with gadolinium contrast, CMR allows better recognition of myocardial fibrosis as regions of late gadolinium enhancement (LGE) [51]. CMR is an alternative to an echocardiogram for quantitative measurements of cardiac functions in patients with poor acoustic windows or who have other limitations rendering the echocardiogram evaluation suboptimal. Though no criteria have been made for the indication of CMR, the 2022 ECS guidelines suggest CMR in newly found PVCs with atypical origins, or in patients with inconclusive echocardiography results, to rule out subtle structural heart diseases [52]. Generally, the most common indication for CMR is diagnosis followed by risk stratification. The morphology and extent of LGE help differentiate non-ischemic fibrosis from scarring related to myocardial infarction and can be used to detect subclinical atherosclerosis in asymptomatic patients [51]. Most patients with ischemic cardiomyopathy have significant scars compared to PIC patients who have none or minimal scarring in a non-ischemic pattern that could help confirm PVC origins [44,47].

Other specific investigations could also be considered when other causes, such as infections or endocrinopathies, are suspected [43]. Genetic cardiomyopathies can present with arrhythmias as their first presentation and AHA/ACC guidelines recommend genetic testing be done in selected cases to find dilated cardiomyopathy (DCM), hypertrophic cardiomyopathy (HCM), and arrhythmic cardiomyopathy (ACM) [51,53]. Clues that suggest underlying cardiomyopathy are multifocal PVCs, PVCs with RBBB pattern (LV origin), or previous family history [51]. It is to be noted that all of these are genetic diseases related to cardiomyopathies that might present with PVCs and are not PIC, because these cardiomyopathies are not the result of PVCs. To the date of this review, we have not found specific genes that are related to isolated PVCs. 

**Figure 1:**
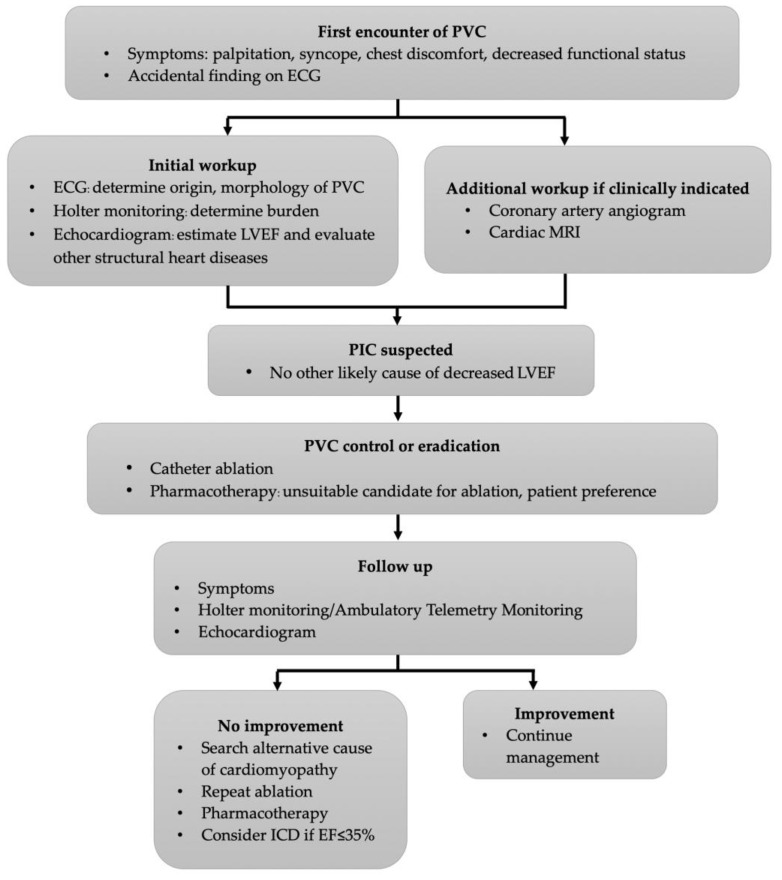
Flow chart suggesting evaluation and management of patients with PVC.

We propose a paradigm of patient evaluation and management, shown in Figure 1.

PIC is LV dysfunction caused by PVC. However, cardiomyopathy itself can also induce PVC [21]. Therefore, it is important to differentiate between these two conditions to select the patient for appropriate treatment. PIC is a diagnosis of exclusion and is characterized by the improvement of LVEF after PVC ablation; however, LV dysfunction still persists in cardiomyopathy-induced PVC after PVC ablation [21]. Several characteristics are suggested to differentiate between these two conditions [2,44]. LV dysfunction in PIC occurs due to the PVC itself rather than a preexisting cardiac condition, so patients with PIC tend to be a healthier, younger population without preexisting heart conditions [2,44]. Echocardiograms could also suggest the etiology, including a lower LVEF in cardiomyopathy-induced PVC compared to PIC [2,44]. Other investigations, such as CMR with late gadolinium enhancement, can indicate cardiomyopathy-induced PVC with more extensive scarring compared to PIC, which has minimal or no scarring [37,44]. Characteristics of PVC can help differentiate PIC from cardiomyopathy-induced PVC. PIC correlates with the PVC burden, although a lower PVC burden can also cause PIC [2,7,8,9,10,11,12,13,14,15,16,20,21,44]. Thus, PIC tends to have a higher PVC burden compared to cardiomyopathy-induced PVC [2,7,8,9,10,11,12,13,14,15,16,44]. Multifocal PVCs are suggestive of cardiomyopathy-induced PVC, compared to monomorphic PVC in PIC [2,8,17,24,44]. The PVC origin from the outflow tract and epicardium favors PIC [2,8,12,16,17,44]. Most studies demonstrating the association between PVC and LV dysfunction reported PVC originating from the outflow tract, and some studies also reported the association between epicardial-origin PVC and cardiomyopathy [8,12,16,17,44]. However, it is crucial to recognize that PVC could also further deteriorate LV dysfunction in preexisting cardiac conditions, and PVC ablation could partially reverse LV dysfunction, suggesting multiple factors contributing to LV dysfunction [2,44]. Table 2 summarizes some key information to differentiate between PIC and cardiomyopathy-induced PVC.

## 5. Treatment

PIC is an important reversible secondary cause of left ventricular (LV) dysfunction. Patients suspected to have PIC should undergo treatment with the primary objective of restoring the deteriorated cardiac function caused by frequent PVCs. It has been shown that, in patients with PIC, LVEF could be improved by reducing or eliminating the PVC burden. In general, the management of PIC includes the search for and treatment of secondary causes of PVCs, catheter ablation to reduce or eliminate PVC burden, or pharmacotherapy to suppress PVCs.

### 5.1. Lifestyle Modification

Lifestyle modification is a readily available method that can be advised for every patient suspected to be suffering from PIC. A few studies have shown that caffeine consumption, smoking, and less physical activity are weakly associated with higher PVC frequency [54,55,56]. Lifestyle modification may be used as an adjunctive initial strategy, but this method alone is not sufficient. The evidence on the effectiveness of modifying these factors to reduce PVC burden is very limited [57]. Co-treatment with other evidence-proven methods, catheter ablation or medication, is necessary to further reduce PVC burden and improve cardiac function.

### 5.2. Catheter Ablation

Catheter ablation is considered the first-line therapy for patients with PIC because of its high success rate and low complication rate [52]. It has received a class I recommendation from the 2022 ESC Guidelines for the management of patients with ventricular arrhythmias and the prevention of sudden cardiac death for the treatment of cardiomyopathy suspected to be caused by frequent and predominately monomorphic PVCs [52].

Results from a retrospective multicenter cohort study showed that the acute procedural success rate of catheter ablation for idiopathic PVCs was achieved in 84% of the patients and the success rate at a mean follow-up time of 1.9 years was 71% without the use of antiarrhythmic medication [30]. Specifically, in the PIC population, catheter ablation could reduce the mean PVC burden from 27% to 5% and could restore the mean LVEF from 38% to 50% [30]. Other studies have found that catheter ablation could reduce the mean PVC burden from 28–29% to 1–3% and resulted in an improvement of LVEF from 37–40% to 48–52% [58,59]. In comparison to the group that used AAD therapy, the proportion of PIC patients who had LVEF normalization was significantly greater in the group that underwent catheter ablation (47% versus 21%) [60].

Previous studies demonstrated LV recovery after ablation in PIC, which was reported within 4–6 months [11,61]. A prospective study in patients with frequent PVC referred for ablation reported that the majority of patients had LV recovery within 4 months [61]. Another study in patients with frequent symptomatic PVC referred for ablation reported LV recovery within 6 months [11]. Compared to atrial fibrillation (AF), previous studies have reported LV recovery after restoration of sinus rhythm within 1 to 3 months [62,63,64]. A study in AF with heart failure reported that LVEF recovery occurred within 1.5 months post-ablation [63].

Despite the excellent outcomes, the major drawback of catheter ablation has always been the risk of potential complications. The total risk of complications has been reported to be around 5.2% and vascular access-related complications were most commonly reported. Major complications accounted for 2.4%, comprising cardiac tamponade (0.8%), pseudoaneurysm (0.7%), hematoma (0.34%), arteriovenous fistula (0.25%), atrioventricular block (0.1%), and others (0.3%) [30].

Regarding the anatomical origin of PVC, some locations are more challenging than others, and this could greatly affect the success and complication rate of catheter ablation. RVOT PVC seems to have the highest success rate and lowest complication rate [30]. On the other hand, catheter ablation of PVCs originating from epicardium, LV summit, intramural, papillary muscles, and para-Hisian tends to be associated with a lower success rate and higher risk of complications [4]. This is due to several reasons, such as the need for a subxiphoid or particular surgical approach, the proximity to a coronary artery, or the anatomic variability and the motion of the structure [4]. The advancement in catheter ablation technology has made it possible to ablate these locations, and the option of catheter ablation should not be ruled out when considering the treatment option of PIC originating from these challenging locations.

In addition to the location of PVC foci, pharmacologic alternatives, patient comorbidities and preferences, and physician experience must all be taken into consideration and discussed with patients to make an informed decision.

### 5.3. Pharmacologic Treatment

Beta blockers and non-dihydropyridine calcium channel blockers (CCBs) are considered first-line therapy in patients with idiopathic PVCs in the absence of structural heart disease or PIC, especially for RVOT and fascicular PVCs [52].

Beta blockers are relatively safe medications that have a long track record of use in management of symptomatic frequent idiopathic PVCs. A randomized placebo-controlled study demonstrated their effectiveness in reducing PVC burden from 24,000 to 16,000 counts per 24 h [65]. However, compared to other classes of AAD, beta blockers have modest effects in the reduction of PVC burden [60,66]. Nevertheless, apart from their role in suppressing PVCs, beta blockers have the additional benefit of lowering the risk of mortality in chronic heart failure management [67].

Non-dihydropyridine CCBs are another group that is frequently used for the management of idiopathic PVC without structural heart disease or cardiomyopathy. However, their negative inotropic effect could further deteriorate the already compromised cardiac function in PIC, making them contraindicated in patients with PIC [52,68].

One retrospective study demonstrated class I and class III AADs to be even more efficacious in reducing PVC burden when compared to beta blockers (mean PVC reduction of 82% versus 36%) [60]. However, the fatal proarrhythmic side effect of class IC AADs limited their use in the post-myocardial infarction population and they are currently generally avoided in the presence of any structural heart disease [67,69]. Recently, a small retrospective study involving 20 patients with suspected PIC with an average of 1.3 failed ablations showed that flecainide and propafenone could suppress the mean PVC burden from 36% to 10% and resulted in an increase in LVEF from 37% to 49% [70]. Over a mean follow-up interval of nearly 4 years, no fatal arrhythmias or mortality were reported [70].

In one randomized controlled trial in patients with congestive heart failure, amiodarone showed an effective reduction in PVCs and significant improvement in LVEF (from 25% to 35%) despite having no benefit on overall survival (even though the result trended in favor of amiodarone among heart failure patients of nonischemic cardiomyopathy [71]. Although amiodarone is effective in reducing PVC burden, improving LVEF, and could be administered safely in severe LV dysfunction, its various systemic organ toxicities have limited its long-term use, especially in young patients [27].

The 2022 ESC Guidelines for the management of patients with ventricular arrhythmias and the prevention of sudden cardiac death recommend AADs for the treatment of cardiomyopathy suspected to be caused by frequent and predominately monomorphic PVCs in cases where “catheter ablation is not desired, suspected to be high-risk, or unsuccessful” [52]. Table 3 summarizes the studies reporting treatment outcomes on PVC and LV dysfunction.

### 5.4. Prognosis

In a retrospective multicenter study of PIC patients who underwent catheter ablation, improvement in LVEF was seen in 85% of PIC patients who underwent ablation, with 67% experiencing more than a 10% increase in LVEF [30]. On the other hand, 15% of PIC patients showed no improvement or even worsening of cardiac function [30].

Several studies have identified the predictors of LV recovery after the elimination of PVCs [2,40,72]. One study showed that the chronic ablation outcome and the absence of preexisting cardiomyopathy were independent predictors of LVEF improvement [2]. After successful PVC elimination, PIC patients with preexisting cardiomyopathy showed improvement in LV function but to a lesser extent when compared to PIC patients without preexisting cardiomyopathy [3]. Deyell et al. discovered that wider PVC QRS duration, regardless of the site of origin, was associated with a lack of LV function normalization after successful ablation [40]. The group hypothesized that PVC QRS duration reflected the underlying fibrosis of the myocardium, which would be less likely to reverse after PVC elimination [40]. Another study found that the myocardial scar mass, as shown in late gadolinium enhancement cardiac magnetic resonance (LGE-CMR), was independently associated with the lack of LVEF improvement after catheter ablation [72].

## 6. Conclusions

PVC is a commonly encountered clinical problem and PIC has gained more recognition by physicians. Currently, PVC burden is the most reliable predictor of development of PIC, although other risk factors such as PVC location, QRS duration of the PVC, and PVC coupling intervals may be beneficial. However, current evidence about these three factors is not strong and may be conflicting. Cardiac imaging tools, such as echocardiograms and cardiac MRIs, are crucial in identifying the cardiac anomalies that could be the underlying cause of cardiomyopathy. Antiarrhythmic drugs, lifestyle modifications and catheter ablation are the cornerstones of the management of this clinical entity. We hope this article increases recognition of PIC among the medical community, which will result in early diagnosis and treatment of patients.

## Figures and Tables

**Table 1:** Clinical studies on PVC burden, characteristics, and PVC-induced cardiomyopathy.

Population	Pattern	PVC-Associated Symptom	Method of PVC Burden Assessment	N/Age/Female/Mean F/U	F/U Duration from PVC to Cardiomyopathy	Main Findings	Ref.
Frequent PVC (>1000/day)	RVOT, LVOT	No	2–3 times Holter monitoring	239/43 ± 13 years/50.6%/5.6 ± 1.7 years	5.6 ± 1.7 years	PVC burden > 20,000/24 h was associated with LVEF decline and increase LVEDd.	[7]
Patients underwent RFA with PVC-associated symptoms	RVOT	Yes	24 h Holter monitoring	40/50 ± 2 years/77.8%/8 ± 1 months	Cross-sectional	PVC burden > 20% was associated with a decrease in LVEF, enlarged LVEDd, LVESd, degree of MR, and a higher NYHA functional class.	[8]
Patients underwent RFA with PVC-associated symptoms	Any	Yes	24 or 48 h Holter monitoring	70/42 ± 17 years/57%/-	Cross-sectional	PVC burden > 10,000 or >20,000/24 h was associated with a decline in LVEF. PVC burden > 10% or >20% was associated with LVEF decline and increase LVEDd. Increased PVC duration > 140 ms was associated with a lower LVEF. The threshold for PVC burden associated with LVEF decline for RV and LV origin PVC was 10% and 20%, respectively.	[9]
Patients underwent RFA	Any	Any	24 h Holter monitoring	174/48 ± 13 years/50%/-	Cross-sectional	PVC burden > 24% was a predictor of impaired LVEF with a sensitivity of 79% and specificity of 78% (AUC 0.89). There was no difference in RV and LV origin PVC for PVC-induced cardiomyopathy.	[11]
Frequent PVC > 10/h underwent RFA with symptomatic PVC-associated	Any	Yes	24 h Holter monitoring	60/45 ± 11 years/63.3%/6 months	Cross-sectional	The LVEF, LVESd, and LVEDd were correlated with PVC burden.	[10]
Frequent PVC > 10/h	RVOT	-	24 h Holter monitoring	108/50 ± 16 years/69%/-	Cross-sectional	Higher PVC burden was associated with LV dysfunction. PVC > 10,000/24 h was an independent predictor of LV dysfunction.	[12]
Frequent PVC referred for RFA	RVOT	Any	24 h Holter monitoring	27/47 ± 15 years/59.3%/-	Cross-sectional	There was no difference in PVC/24 h between impaired and normal LVEF.	[17]
Frequent PVC referred for RFA	Any	Any	24 h Holter monitoring	51/49 ± 15 years/27.5%/-	Cross-sectional	PVC interpolation was associated with a higher PVC burden and impaired LVEF.	[13]
Frequent PVC	Any	Any	24 h Holter monitoring	183/49 ± 15 years/59.4%/-	Cross-sectional	PVC burden was associated with cardiomyopathy, but not the total heart rate. PVC burden > 16% was a predictor of cardiomyopathy with a sensitivity of 100% and specificity of 87% (AUC 0.96).	[14]
Frequent PVC referred for RFA	Any	Any	24 h Holter monitoring	241/48 ± 14 years/52%/-	Cross-sectional	Asymptomatic, longer palpitation duration (>30 months), and PVC burden were independent predictors of PVC-induced cardiomyopathy.	[15]
Frequent PVC referred for RFA	Any	Any	24 h Holter monitoring	294/48 ± 14 years/46.6%/-	Cross-sectional	PVC QRS duration, PVC epicardial origin, PVC burden, and symptom duration were independent predictors of cardiomyopathy. Broad PVC QRS was associated with a lower PVC burden threshold for cardiomyopathy. PVC QRS duration > 150 ms was a predictor of cardiomyopathy with a sensitivity of 80% and specificity of 52% (AUC 0.66).	[16]
PVC ≥ 10%/24 h underwent ablation with normal baseline LVEF	Any	Any	24 h Holter monitoring	45/53 ± 16.5 years/50%	Median 14 (8–32) months	PVC QRS duration ≥ 153 ms and non-outflow tract origin were predictors of LV dysfunction. There was no association with PVC burden.	[22]

AUC, area under the curve; LVEDd, left ventricular end-diastolic dimension; LVEF, left ventricular ejection fraction; LVESd, left ventricular end-systolic dimension; MR, mitral regurgitation; NYHA, New York Heart Association; RFA, radiofrequency ablation.

**Table 2:** Comparison of characteristics of PVC-induced cardiomyopathy (PIC) versus cardiomyopathy-induced PVC.

	PVC-Induced Cardiomyopathy (PIC)	Cardiomyopathy-Induced PVC
PVC morphology	Monomorphic, smooth contours with sharp QRS complex	Multifocal, broad notching, and slurred QRS
PVC burden	Higher	Lower
Origin	RVOT, LVOT	Non-outflow tracts
Echocardiogram	Global hypokinesia	Regional hypokinesia
CMR	Absent or minimal myocardial fibrosis	Significant fibrosis

CMR, cardiac magnetic resonance imaging.

**Table 3:** RFCA and AAD outcome of patients with frequent PVC and LV dysfunction.

Intervention	Patients with LV Dysfunction	Presence of Concomitant SHD	PVC Burden (Initial)	PVC Burden (Final)	LVEF (Initial)	LVEF (Final)	Ref.
RFCA	40	None	20%	N/A	38%	49%	[72]
RFCA	171	48% ICM	24%	N/A	39%	42% (If successful ablation)	[73]
RFCA	67	100% ICM	29%	4.6%	34%	42%	[74]
RFCA	120	11% ICM 13% NICM	25%	4.6%	41%	59%	[29]
RFCA	39	28% ICM	21%	N/A	44%	52%	[75]
RFCA	215	37% SHD	23%	1%	35%	44%	[76]
RFCA	54	19% ICM 19% NICM	28%	0.8%	40%	52%	[58]
RFCA	77	23% ICM 18% DCM 9% VHD	28%	3.6%	37%	49%	[59]
65% Flecainide 35% Propafenone	20	N/A	36%	10% (Overall) 11% (Flecainide) 7% (Propafenone)	37%	49% (Overall) 49% (Flecainide) 48% (Propafenone)	[70]
RFCA	31	None	23%	N/A	35%	42%	[77] *
RFCA	55	None	25%	0.4% (If successful ablation)	35%	50%	[78]
RFCA	96	23% ICM 8% DCM 3% VHD	26%	4% (Overall) 1% (If successful ablation)	38%	50% (Overall) 54% (If successful ablation)	[79]
RFCA	66	17% ICM 5% NCCM 2% VHD	21%	N/A	28%	42%	[80]
RFCA	30	100% NICM	23%	4.7% (Overall) 1% (If successful ablation)	38%	45%	[5]
RFCA	245	None	27%	5%	38%	50%	[30]
68% RFCA 32% AAD (4% Flecainide, 5% Propafenone, 23% Amiodarone)	57	None	30%	<6%	35%	67% LVEF normalization 33% persistent LV dysfunction	[81] *
44% RFCA 56% AAD	121	83% DCM 17% ICM	23%	9.2% (Overall) Reduction of 25,366 and 12,278 PVC/day in RFCA and AAD group, respectively	37%	42% (Overall) LVEF normalized in 47% and 21% of patients in RFCA and AAD group, respectively	[60]
RFCA	75	None	26%	2%	39%	59%	[61] *
RFCA	65	65% DCM 35% ICM	10–20% (41%) 20–30% (31%) >30% (28%)	N/A	26%	33%	[82]
RFCA	24	38% DCM 38% ICM	15%	1%	32%	43%	[83]
RFCA	37	None	29%	N/A	37%	18% increase (70%) 0% increase (30%)	[40] *
RFCA	69	29% NICM	29%	3.5%	35%	46%	[2]
Ivabradine	35	100% DCM	18%	10.5%	33%	38%	[84]
RFCA	57	N/A	33%	1.9%	35%	54%	[10]
RFCA	15	100% ICM	22%	2.6%	38%	51%	[6]
RFCA	6	None	17,717 PVC/day	268 PVC/day	42%	57%	[85]
RFCA	22	None	37%	0.7% (If successful ablation)	34%	53%	[11]
RFCA	8	None	17,541 PVC/day	2054 PVC/day (Overall) 507 PVC/day (If successful ablation)	39%	59% (Overall) 62% (If successful ablation)	[17]
Amiodarone	336	72% ICM 28% NICM	254 PVC/hour	44 PVC/hour	25%	35%	[71]

* Study included only patients who underwent successful ablation, AAD, antiarrhythmic drug; DCM, dilated cardiomyopathy; ICM, ischemic cardiomyopathy; LV, left ventricle; LVEF, left ventricular ejection fraction; N/A, not available; NCCM, noncompaction cardiomyopathy; NICM, non-ischemic cardiomyopathy; RFCA, radiofrequency catheter ablation; SHD, structural heart disease; VHD, valvular heart disease.

## Data Availability

Data sharing is not applicable.
